# Respiratory pyramid interface (RPI): a conceptual three-dimensional pyramid model for visualization of lung mechanics during mechanical ventilation

**DOI:** 10.3389/fphys.2026.1846417

**Published:** 2026-07-29

**Authors:** Serdar Efe

**Affiliations:** Department of Internal Medicine, Division of Intensive Care, Bursa Uludağ University Faculty of Medicine, Bursa, Türkiye

**Keywords:** expiratory time constant, intensive care, mechanical power, mechanical ventilation, respiratory mechanics, visual interface

## Abstract

Mechanical ventilation is a life-saving intervention in critically ill patients; however, the real-time integration of its key physiological determinants elastic load, resistive load, and obstructive components remains cognitively demanding at the bedside. Current monitoring approaches present these parameters in fragmented formats, which may limit rapid interpretation. The Respiratory Pyramid Interface (RPI) is a conceptual, hypothesis-generating framework that integrates the fundamental variables of respiratory mechanics flow (F), pressure (P), and volume (V) into a unified three-dimensional geometric model. Within this structure, resistive, elastic, and obstructive components are represented along the pyramid edges, while derived indices including mechanical power (MP), driving pressure (DP), static compliance (Cstat), the Pyramid Asymmetry Index (PAI), and a pressure-based surrogate of the expiratory time constant (τ_est) are dynamically mapped. The framework was demonstrated across eleven simulated pathophysiological scenarios, each producing distinct geometric patterns corresponding to specific mechanical phenotypes. Importantly, RPI does not introduce new physiological variables but offers a structured visual integration of routinely available ventilator data, intended to support hypothesis generation, education, and pattern recognition rather than to provide direct treatment guidance. Limitations of the underlying single-compartment model including regional lung heterogeneity, pendelluft, spontaneous respiratory effort, and elevated neural respiratory drive are acknowledged. Prospective validation is required before the proposed indices can be considered for clinical decision support.

## Introduction

Mechanical ventilation is a cornerstone of critical care medicine, yet its safe and individualized application demands continuous integration of multidimensional physiological information. Clinicians must simultaneously interpret airway pressures, tidal volumes, flow patterns, and time-dependent respiratory mechanics while adjusting ventilator settings to support lung protection and adequate gas exchange (Slutsky and Ranieri, 2013; Papazian et al., 2019).

Despite significant advances in ventilator technology, current interfaces continue to present respiratory variables as spatially and temporally fragmented outputs: separate numerical readouts, scalar waveforms, and pressure–volume or flow–volume loops displayed in isolation. Several modern ventilator platforms have introduced graphical lung representations integrated with adaptive ventilation modes (Arnal et al., 2008; Sulemanji et al., 2009), yet most are mode-dependent or vendor-specific and do not provide an integrated view of the dominant mechanical phenotype. The fragmentation of available data imposes substantial cognitive burden, particularly during high-acuity situations or for less experienced clinicians, and may delay recognition of critical patterns such as ventilator-induced lung injury (VILI) risk, patient–ventilator asynchrony, or dynamic hyperinflation (Tobin et al., 2010; Blanch et al., 2015). The limitation in current practice is therefore not the availability of physiological data, but the clinician’s ability to rapidly integrate multiple variables into coherent, actionable patterns under time pressure.

Mechanical power (MP) the energy delivered to the respiratory system per unit time has emerged as a unifying metric of VILI risk, encompassing elastic, resistive, and frequency-dependent components (Gattinoni et al., 2016; Becher et al., 2019). However, its bedside adoption has been limited by the absence of an intuitive display framework. The expiratory time constant (RCexp), governing lung-emptying dynamics and auto-PEEP propensity, similarly remains underutilized (Marini, 2011; Depta et al., 2024).

**Figure 1:**
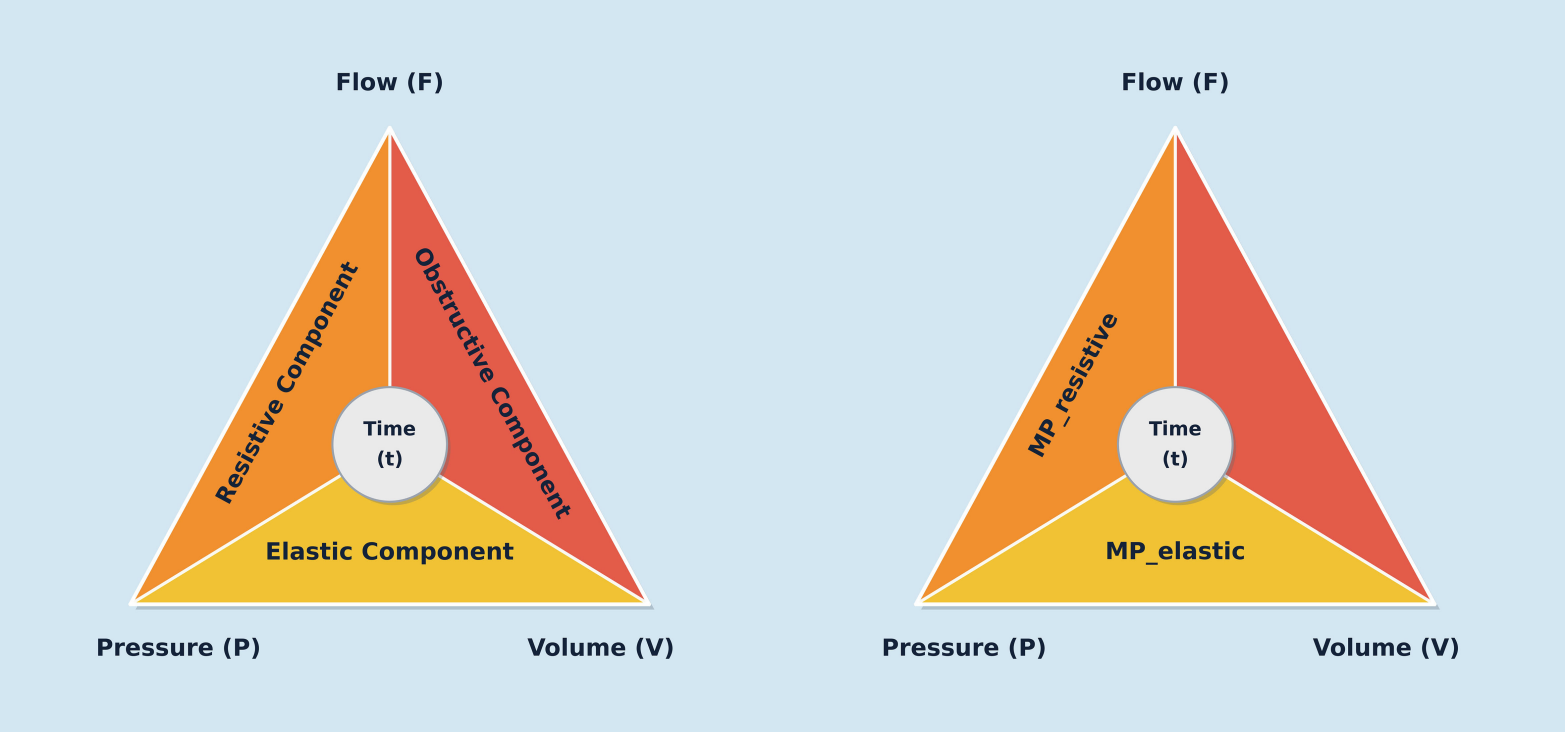
Conceptual structure and mechanical-power decomposition of the Respiratory Pyramid Interface (RPI). Upper panel: the triangular framework represents the integration of respiratory mechanics, with each edge corresponding to elastic (P–V), resistive (P–F), and obstructive (F–V) components; time acts as a central coordinating parameter. Lower panel: mechanical power is decomposed into elastic (MP_el) and resistive (MP_res) components mapped onto the pyramid edges.

I introduce the Respiratory Pyramid Interface (RPI), a conceptual, software-based visual framework that integrates elastic, resistive, and obstructive respiratory components into a unified triangular geometric structure (**Figure 1**). I additionally propose: (1) τ_est, a pressure-derived surrogate of the expiratory time constant requiring no direct flow measurement; and (2) the Pyramid Asymmetry Index (PAI), a ratio of resistive to elastic (driving) pressure describing the dominant mechanical phenotype. Both indices are presented as integrative descriptors rather than novel standalone metrics, and as a basis for hypothesis generation rather than treatment guidance.

## Nomenclature

To standardise notation, all symbols used in the equations below are defined here. P, airway pressure (cmH2O); Ppeak, peak airway pressure (cmH2O); Pplat, plateau pressure (cmH2O); PEEPtot, total positive end-expiratory pressure (cmH2O); VT, tidal volume (L); RR, respiratory rate (breaths·min^-^¹); Flow, inspiratory flow (L·s^-^¹); Tinsp, inspiratory time (s); Texp, expiratory time (s); R, airway resistance (cmH2O·L^-^¹·s); Cstat, static respiratory-system compliance (mL·cmH2O^-^¹); DP, driving pressure = Pplat − PEEPtot (cmH2O); MP, mechanical power (J·min^-^¹); MP_res and MP_el, resistive and elastic components of mechanical power (J·min^-^¹); PAI, Pyramid Asymmetry Index (dimensionless); RCexp, expiratory time constant (s); τ_est, pressure-based estimate of the expiratory time constant (s). Subscripts are used consistently throughout the text, equations, and figure captions.

## Conceptual design of the respiratory pyramid interface

The RPI is structured as a triangular geometric framework in which each edge encodes a fundamental respiratory relationship. The elastic edge (P–V) reflects static compliance and elastic work of breathing. The resistive edge (P–F) encodes airway resistance and the resistive pressure gradient (Ppeak − Pplat). The obstructive edge (F–V) provides insight into expiratory flow limitation, air trapping, and dynamic hyperinflation. Each edge is color-coded according to a severity gradient from purple (normal) through yellow and orange to red (critical) intended to facilitate visual discrimination of the dominant pathophysiological load. Together, the three edges reconstruct the classical equation of motion of the respiratory system, as expressed in Equation 1:

(1) P = (VT/Cstat) + (R × Flow) + PEEPtot

Time is incorporated as a central coordinating dimension, linking scalar waveforms with loop-based representations. Within this conceptual framework, clinicians may transition from fragmented waveform analysis toward integrated pattern recognition within a single visual field.

## Mechanical power decomposition

Within the RPI framework, mechanical power is decomposed into elastic and resistive components using a simplified bedside formulation derived from Gattinoni et al. (2016):

(2) MP_res = 0.098 × RR × VT × (Ppeak − Pplat)

(3) MP_el = 0.098 × RR × 12 × VT × (Pplat − PEEPtot)

The constant 0.098 converts the product of pressure and volume from cmH2O·L into joules(since 1 cmH2O = 98.0665 Pa and 1 L = 10^-^³ m³, 1 cmH2O·L ≈ 0.0981 J); with RR expressed per minute, mechanical power is therefore reported in J·min^-^¹. These components are visually encoded in the resistive and elastic edges of the pyramid, respectively, allowing visual comparison of their relative magnitudes. (Equations 2,3) follow the geometric mechanical-power decomposition of Gattinoni et al. (2016), in which the resistive component corresponds to the rectangular pressure–volume area (Ppeak − Pplat) × VT and the elastic component to the triangular area ½ × (Pplat − PEEPtot) × VT; the static PEEP–volume term (PEEPtot × VT) is omitted, as the framework focuses on the dynamic tidal energy delivered per breath; they are intended for visual–conceptual integration only and do not replace full energy-based mechanical-power analysis (González-Castro et al., 2024).

## Pyramid asymmetry index

The Pyramid Asymmetry Index is a dimensionless ratio that summarises the relative balance between resistive and elastic (driving) pressure, as defined in Equation 4:

(4) PAI = (Ppeak − Pplat)/(Pplat − PEEPtot)

A higher PAI reflects a greater relative resistive contribution (as in severe bronchospasm or COPD), whereas a lower PAI reflects predominance of the elastic load (as in ARDS or pulmonary fibrosis). PAI thus provides a single numerical summary of the pyramid shape and the relative weighting of resistive and elastic pressure. It is presented as a continuous descriptor without fixed diagnostic cut-offs, and it additionally serves as the phenotype term from which the pressure-based time-constant estimate is derived (Equation 6).

Although PAI summarises the resistive–elastic balance, it does not distinguish between diseases with similar mechanical balance but fundamentally different pathophysiology and therapeutic implications. For example, despite comparable PAI values, optimal ventilatory strategies for recruitable ARDS and non-recruitable fibrotic lungs may differ substantially (Nolan et al., 2025). PAI should therefore be interpreted as a mechanical descriptor rather than a disease-specific treatment guide, and always in conjunction with clinical context, imaging, and gas-exchange assessment. It is presented as an integrative descriptor for hypothesis generation; it has not been validated against patient-centred outcomes and should not be used as a standalone tool for treatment guidance.

## Pressure-based estimation of the expiratory time constant (τ_est)

The classical expiratory time constant is defined in Equation 5 as:

(5) RCexp = R × C

Under passive, volume-controlled ventilation with approximately constant inspiratory flow, resistance can be expressed as R = (Ppeak − Pplat)/Flow and compliance as C = VT/(Pplat − PEEPtot). Because constant inspiratory flow implies Flow ≈ VT/Tinsp, substitution yields:

(6) τ_est = Tinsp × (Ppeak − Pplat)/(Pplat − PEEPtot) = Tinsp × PAI

**Figure 2:**
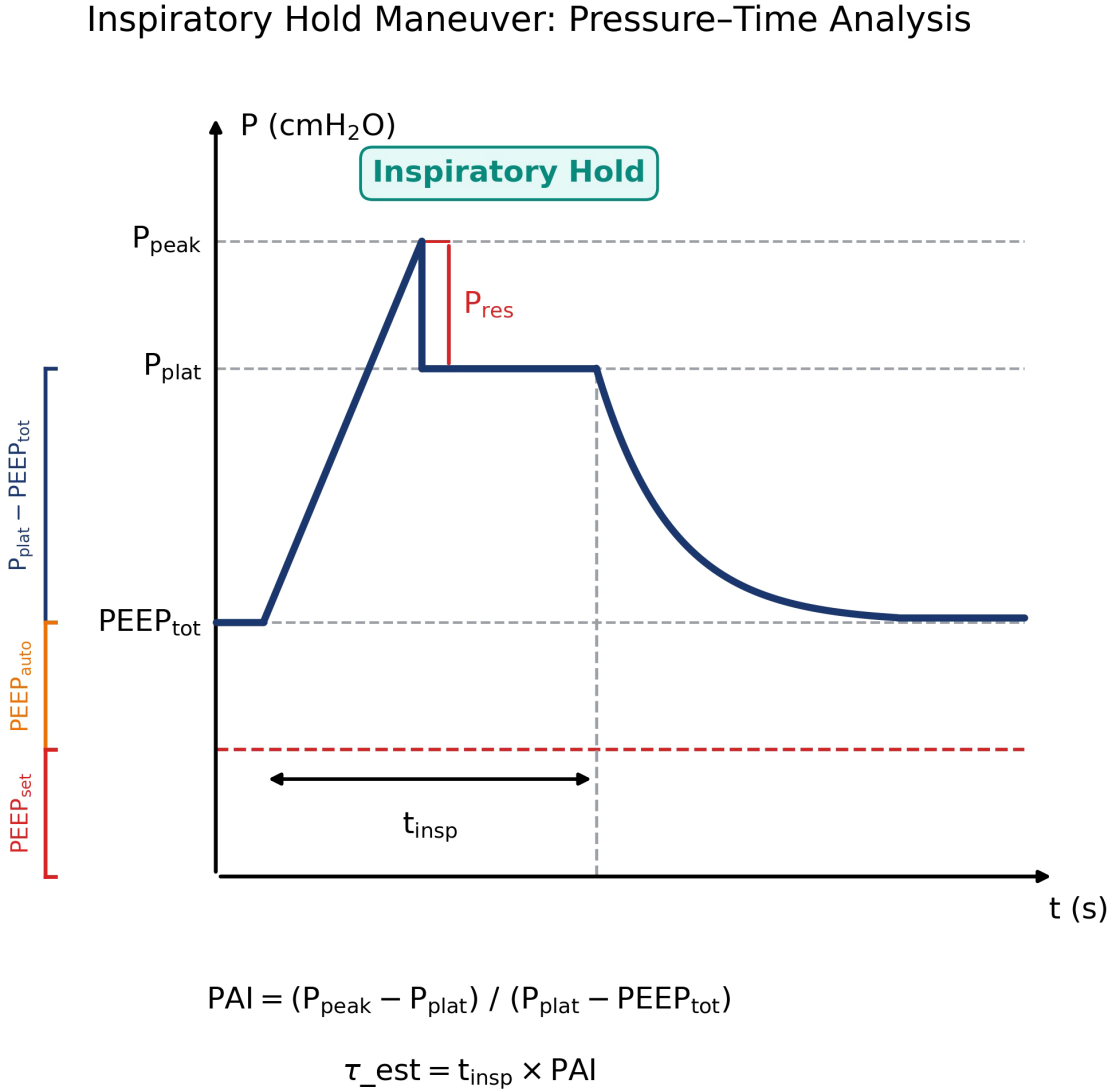
Inspiratory hold manoeuvre and airway-pressure decomposition. Schematic pressure–time tracing of a passive volume-controlled breath with an end-inspiratory hold, showing peak pressure (Ppeak), plateau pressure (Pplat), total PEEP (PEEPtot) and set PEEP (PEEPset); the resistive pressure drop (Pres = Ppeak − Pplat), intrinsic PEEP (PEEPauto), the driving-pressure span (Pplat − PEEPtot) and inspiratory time (Tinsp). This decomposition enables bedside calculation of the Pyramid Asymmetry Index, PAI = (Ppeak − Pplat)/(Pplat − PEEPtot), and the pressure-based expiratory time-constant estimate, τ_est = Tinsp × PAI.

This formulation eliminates the need for direct flow measurement by expressing both resistance and compliance in terms of pressure ratios and inspiratory-time parameters routinely displayed on modern ventilators (**Figure 2**). As shown in (Equation 6), τ_est corresponds to the product of the inspiratory time and PAI and recovers the intrinsic respiratory time constant (R × C).

### Derivation and interpretation of τ_est thresholds

The proposed interpretive ranges are aligned with published reference values for the single-compartment expiratory time constant (Lourens et al., 2000; Depta et al., 2025). The thresholds restrictive< 0.5 s, normal 0.5–0.8 s, and obstructive 0.8–1.2 s reproduce the established short/normal/long classification, in which reference values for adults with preserved lung function cluster at 0.5–0.8 s (mean ~0.6 s), values above approximately 0.7–0.8 s indicate increased resistance, and markedly prolonged values are characteristic of COPD, asthma, and bronchospasm. The additional > 1.2 s category flags a high auto-PEEP propensity. The 1.2 s threshold should be considered a pragmatic visual categorization boundary rather than a validated physiological cut-off. This is grounded in the exponential lung-emptying relationship, in which approximately 63%, 86%, 95%, and 98% of the tidal volume are exhaled after one, two, three, and four time constants, respectively; near-complete passive emptying therefore requires an expiratory time of roughly three to four time constants. The supplementary criterion for identifying insufficient expiratory time is summarized in Equation 7:

(7) Texp/(3 × τ_est) < 1

operationalises this principle, serving as a practical visual indicator that the available expiratory time is insufficient for approximately 95% emptying and that dynamic hyperinflation is likely. As with PAI, τ_est is intended as an integrative descriptor and not as a standalone treatment-guiding measurement; its thresholds are conceptual and require prospective validation.

## Interactive simulation framework

An interactive simulation environment was developed as a standalone web-based application requiring no installation or server infrastructure. Implemented in client-side HTML5/JavaScript, the framework renders three synchronous waveforms (pressure–time, flow–time, volume–time) alongside selectable loop views (P–V, F–V, F–P) and updates the pyramid canvas in real time to reflect changes in elastic, resistive, and obstructive loads, with color-coded severity and overlaid numerical annotations for MP_el, MP_res, PAI, τ_est, and driving pressure.

Algorithmic generation of the pyramid visualization. We note that, in the current implementation, the indices are encoded through regional colour gradients rather than variable edge lengths; we clarify this mapping explicitly below. The pyramid is rendered on a fixed-size HTML5 canvas (320 × 365 px) as an isosceles triangle whose three vertices remain at constant coordinates in every simulation apex F (Flow) at the top, P (Pressure) at the lower left, and V (Volume) at the lower right so that the geometry itself (vertex positions and edge lengths) does not vary with the patient’s mechanics. Diagnostic information is conveyed not by changing edge lengths but by two superimposed layers. First, the triangle is divided into three sub-regions sharing a common centroid, and each sub-region is filled with a colour drawn from a four-level ordinal palette (lavender → yellow → orange → red, corresponding to severity levels 0–3). The base sub-region (edge P–V) is coloured by MP_el, the left sub-region (edge P–F) by MP_res, and the right sub-region (edge F–V) by the auto-PEEP–derived obstructive load; each continuous value is first computed from Equations (2)–(3) and then mapped to a severity level by fixed thresholds (MP_el:<13, 13–15, >15–17, >17 J·min^-^¹; MP_res:<3, 3–5, >5–7, >7 J·min^-^¹; auto-PEEP: 0, 1–4, 5–8, >8 cmH_2_O), with level 0 (lavender) denoting the normal state so that a fully normal mechanical profile yields a uniformly lavender pyramid. Second, the computed magnitudes (MP_el, MP_res, and, during a hold manoeuvre, Pplat, DP, and Cstat) are printed as numerical labels at the corresponding sub-region centroids. The dimensionless indices PAI (= (Ppeak − Pplat)/(Pplat − PEEPtot)) and τ_est (= Tinsp × PAI) are not mapped to the geometry or to a colour; consistent with their presentation as continuous descriptors without fixed cut-offs, they are reported as numerical values in adjacent fields. This explicit specification fixed vertex coordinates, three colour-mapping rules with the stated thresholds, and centroid-anchored numerical labels allows the visualization to be reproduced independently of the source code provided in Supplementary File S1. A step-by-step pseudocode listing and a corresponding flowchart are provided in Supplementary File S2.

**Figure 3:**
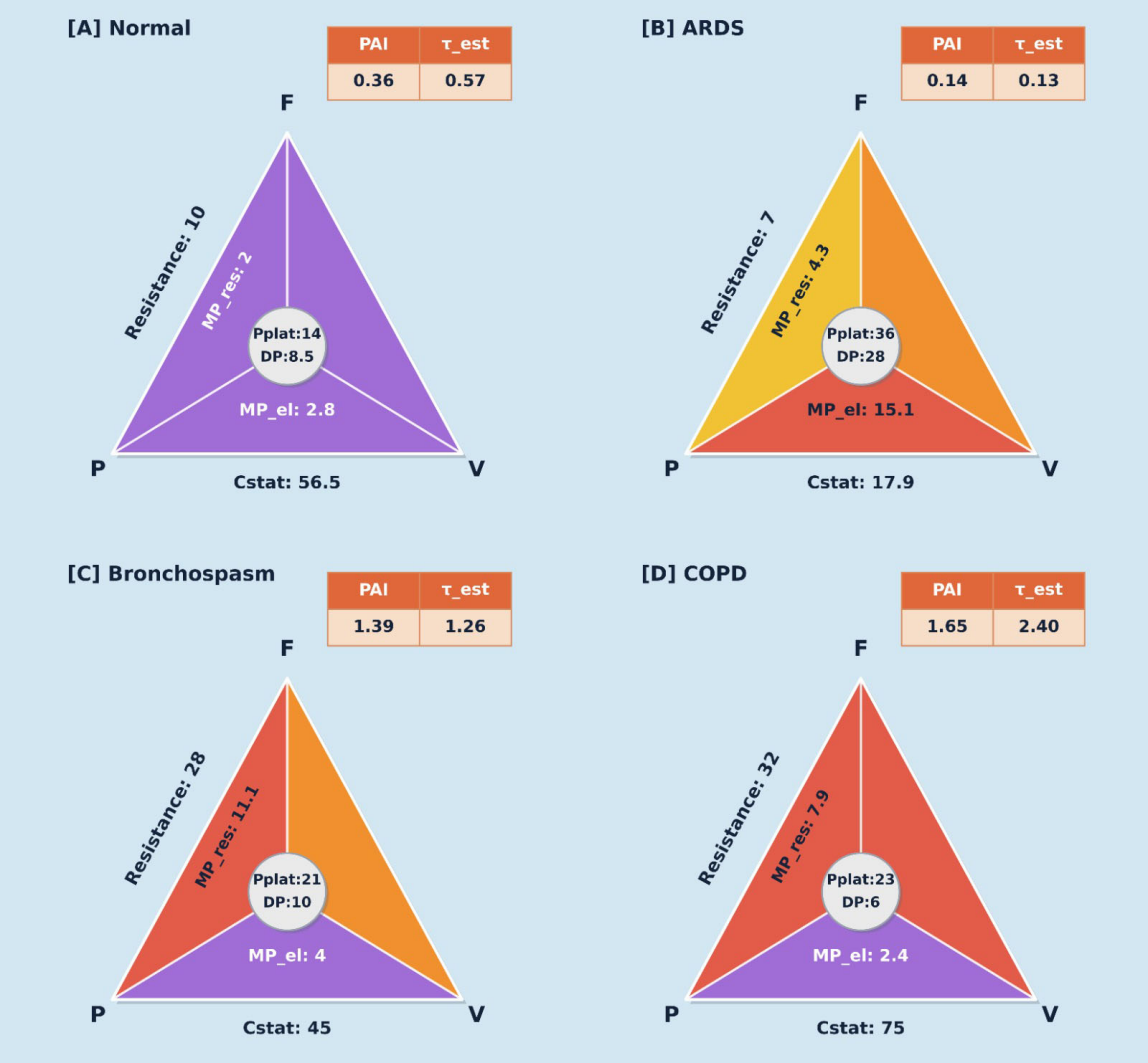
Representative Respiratory Pyramid patterns across four mechanical phenotypes: **(A)** normal mechanics, **(B)** ARDS, **(C)** bronchospasm, and **(D)** COPD with dynamic hyperinflation. In each panel the triangle is divided into three sub-regions resistive (MP_res, left edge), obstructive/auto-PEEP (right edge), and elastic (MP_el, base) each colour-coded by ordinal severity level (lavender → yellow → orange → red). Airway resistance is annotated outside the left edge; plateau pressure (Pplat), driving pressure (DP) and static compliance (Cstat) are reported for each phenotype, and the dimensionless indices PAI and τ_est are tabulated above each pyramid.

Eleven predefined clinical scenarios spanning conditions commonly encountered in intensive care practice are incorporated. Each scenario produces a distinct and reproducible pyramid pattern, intended to facilitate recognition of pathophysiological archetypes through geometric pattern recognition rather than sequential numerical analysis (**Figure 3**). The simulation additionally incorporates six patient–ventilator asynchrony archetypes displayed as annotated waveform panels (de Bie et al., 2025), along with inspiratory and expiratory hold-manoeuvre visualization. Ventilatory parameters and derived indices for all scenarios are provided in **Supplementary Table 1**. The complete interactive implementation is available as Supplementary File S1, and may also be integrated into existing lung-physiology and pathophysiology simulators to support research and educational applications.

## Discussion

The RPI introduces no new physiological variables. All derived metrics are computed exclusively from parameters routinely displayed on modern ventilators: Ppeak, Pplat, PEEP, VT, RR, and Tinsp. The framework is therefore implementable as software on existing platforms without additional sensors or hardware a feature of particular relevance in resource-limited settings or during sensor-calibration interruptions.

### Comparison with existing ventilator graphical interfaces

Several modern ventilator platforms have introduced graphical lung representations that display compliance, resistance, and obstructive components including Hamilton Medical’s Ventilation Cockpit and dynamic lung visualization integrated with Adaptive Support Ventilation (Arnal et al., 2008; Sulemanji et al., 2009), Dräger Smart Pulmonary View, and analogous decision-support displays from other manufacturers. While these interfaces have advanced bedside intuition, they are typically vendor-specific, frequently linked to proprietary adaptive ventilation modes, and tend to emphasize individual mechanical parameters rather than the integrated geometric phenotype. The RPI is intended as a vendor-independent conceptual layer that may complement existing graphical displays by reorganizing routinely available data into a single phenotype-oriented visualization. It is not proposed as a replacement for established ventilator interfaces or for direct treatment guidance.

### Clinical relevance beyond parameter availability

A key consideration is that the limitation in current monitoring is not the absence of measurable physiological parameters, but the fragmentation of their representation. Airway resistance, compliance, and expiratory time constants are accessible through standard ventilator manoeuvres or automated calculations. However, these variables are typically distributed across separate numerical displays, waveforms, and loops, requiring real-time cognitive integration by the clinician. The RPI addresses this gap by transforming dispersed physiological data into a unified geometric representation, intended to facilitate rapid recognition of the dominant mechanical pattern. The proposed indices (PAI and τ_est) should be interpreted as integrative descriptors rather than novel standalone metrics. Their primary role is to assist visual interpretation of relative mechanical contributions, not to replace established measurements or to guide therapy independently.

The present study is intentionally conceptual and hypothesis-generating. It does not aim to replace existing monitoring tools or introduce new physiological measurements, but rather to propose a structured visual framework for integrating routinely available data. The central hypothesis is that transforming multidimensional respiratory information into an intuitive geometric model may facilitate pattern recognition and reduce cognitive load during ventilator assessment. This hypothesis is testable and provides a clear direction for future research, including prospective clinical validation, usability analysis, and evaluation of decision-making performance in simulated and real-world settings.

## Strengths

The RPI integrates routinely available ventilator parameters into a single geometric framework without requiring additional hardware. The pressure-based time-constant estimator (τ_est) reduces dependence on flow sensors. The PAI provides a concise summary of elastic and resistive pressure contributions. Across eleven simulated clinical scenarios, the RPI generates distinct visual patterns that may facilitate rapid pathophysiological differentiation in educational and research settings.

## Limitations

The RPI is built on a single-compartment, passive respiratory model and inherits all of its well-recognized limitations. Real ARDS lungs are markedly heterogeneous, with simultaneous coexistence of aerated, poorly aerated, and consolidated regions; consequently, global pressure–volume measurements may not reflect regional stress and strain, and a single PAI or τ_est value cannot capture intra-pulmonary mechanical heterogeneity, regional pendelluft, or tidal recruitment–derecruitment phenomena (Kuhn and Barjaktarevic, 2024). In patients with strong spontaneous respiratory effort, elevated neural respiratory drive, or ineffective triggering, airway pressure no longer reflects total transpulmonary load, and patient self-inflicted lung injury (P-SILI) may develop without being detected by pyramid-based descriptors (Goligher et al., 2020; Marongiu et al., 2024). The accuracy of τ_est further depends on passive ventilation, approximately constant inspiratory flow (as in volume-controlled ventilation), and the applicability of a single-compartment model assumptions that are frequently violated during pressure-controlled or spontaneous-assisted modes.

Critically, both PAI and τ_est are derived from the plateau pressure (Pplat), and their validity therefore depends on a reliable end-inspiratory occlusion. In routine practice, Pplat is frequently imprecise owing to incomplete airway occlusion, spontaneous inspiratory effort or patient–ventilator asynchrony during the inspiratory pause, circuit or cuff leaks, persistent end-inspiratory flow, and inter-operator variability. Because PAI = (Ppeak − Pplat)/(Pplat − PEEPtot), even modest errors in Pplat propagate non-linearly into both indices and may alter the apparent phenotypic classification, particularly for values close to a threshold boundary. The proposed descriptors should therefore be interpreted only when a stable, reproducible plateau can be obtained under passive conditions, and any borderline classification should be corroborated by repeated measurement together with clinical context, imaging, and gas exchange.

PAI is presented as a continuous descriptor without fixed diagnostic cut-offs and has not been validated against patient-centred outcomes. Importantly, two diseases with comparable PAI or τ_est values may require fundamentally different ventilatory strategies. Oversimplified visual classification therefore carries a real risk of premature or inadequate clinical interpretation, and PAI/τ_est must always be considered together with clinical context, imaging, and gas exchange. The RPI itself has not been evaluated in real-time clinical environments, and its proposed benefits require prospective usability and clinical validation. No conclusions can be drawn regarding patient-centred outcomes at this stage. Furthermore, the proposed indices have not been validated against flow-derived measurements, expert waveform interpretation, clinical outcomes, or external datasets.

## Future directions

Prospective validation of τ_est against flow-derived RCexp across clinical populations including ARDS, obstructive disease, and weaning represents the primary research priority. Usability studies assessing cognitive workload, pattern-recognition performance, and time-to-decision in simulated scenarios, using validated instruments such as the NASA Task Load Index, would provide empirical support for the cognitive-load-reduction hypothesis. Integration of RPI into existing lung physiology/pathophysiology simulators and into ventilator software platforms or bedside monitoring systems could enable real-time pyramid rendering from live data streams and may enhance educational and research applications. Machine-learning-assisted classification of pyramid configurations (Rietveld et al., 2025) could further automate recognition of pathophysiological archetypes, although such applications remain firmly within the realm of future investigation.

## Conclusion

The Respiratory Pyramid Interface introduces a unified geometric framework for integrating elastic, resistive, and obstructive respiratory mechanics into a single conceptual structure. By combining pyramid visualization with a pressure-derived expiratory-time-constant estimator and a Pyramid Asymmetry Index, RPI reorganizes routine ventilator data into an integrated visual interpretation framework. Across eleven simulated clinical scenarios, the framework generated distinct visual patterns consistent with the underlying simulated physiological conditions. RPI is presented as a conceptual, hypothesis-generating tool. Prospective validation is required before the proposed indices and the framework as a whole can be considered for clinical decision support.

## Data Availability

No datasets were generated or analyzed during this study. This Perspective article presents a conceptual framework, and the illustrative patient examples are hypothetical and included solely to demonstrate the proposed methodology.
