# Supplementary material for: Respiratory pyramid interface (RPI): a conceptual three-dimensional pyramid model for visualization of lung mechanics during mechanical ventilation
Source: Front Physiol. 2026 Jul 29;17:1846417. doi: 10.3389/fphys.2026.1846417 (PMC13461443; doi:10.3389/fphys.2026.1846417)
Supplement: Supplementary file 1 [file DataSheet2.pdf]

Respiratory Pyramid Interface (RPI) — Interactive Simulation

Quick-Start Guide · Supplementary File S1

SUPPLEMENTARY FILE S1

Serdar Efe, MD · Dept. of Internal Medicine, Intensive Care Unit · Bursa Uludag University, Bursa, Turkey

Peer Review Version

Respiratory Pyramid — Anatomy & Interactive Zones

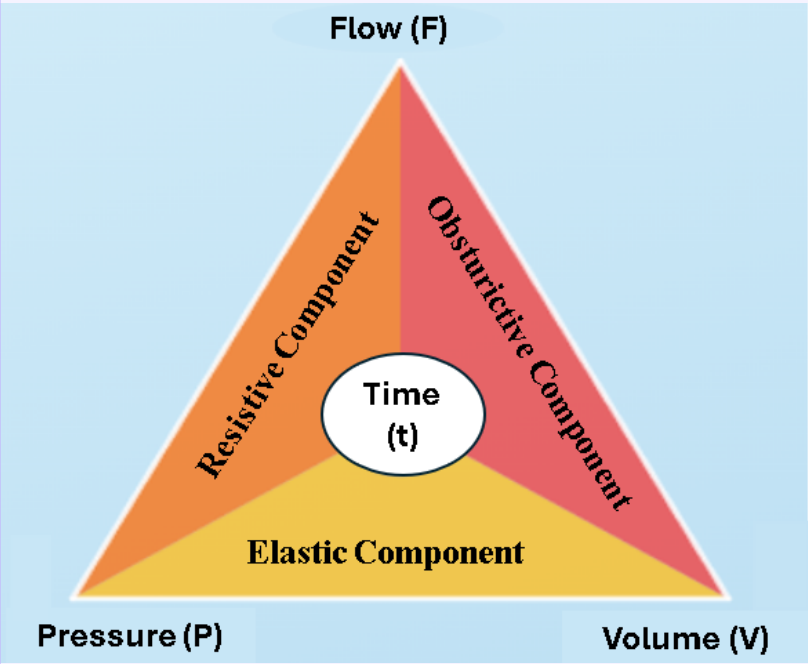

Fig. 1 — Respiratory Pyramid Interface

Zone / Edge	Loop	Physiology	Click Action
**Left edge (F apex → P bottom-left)**	F–P loop	Airway resistance (P_{peak}–P_{plat})	F–P loop Resistive
**Right edge (F apex → V bottom-right)**	F–V loop	Expiratory flow limitation Air trapping	F–V loop Obstructive
**Bottom edge (P bottom-left → V bottom-right)**	P–V loop	Static compliance Elastic work	P–V loop Elastic
**Center — TIME**	—	Time coordinator Links waveforms & loops	Inspiratory Hold (5 sec, auto)
**Right inner zone (inside F–V edge)**	—	Obstructive inner region auto-PEEP zone	Expiratory Hold auto-PEEP display

Clinical Workflow — Step by Step

1

Select a scenario — or enter real patient data manually

→ Click any scenario button in the top row: **Normal, ARDS, COPD, Bronchospasm**, etc. All parameters update instantly.

→ For real patient values, click ■ **Patient Entry** and enter P_{peak}, P_{plat}, PEEP, VT, RR, auto-PEEP, FiO_2, and I:E via the numeric keypad.

→ Confirm that **VCV mode** is selected — required for hold maneuvers.

■ *In the ARDS scenario, inline Prone and VV-ECMO toggles appear — observe how lung mechanics change.*

2

Click the TIME area → trigger the Inspiratory Hold maneuver

→ On the Respiratory Pyramid, click the **TIME** label at the inner center of the triangle.

→ A 5-second inspiratory hold triggers automatically. The waveform freezes at end-inspiration; P_{plat} is displayed.

→ Edge colors update in real time — **any non-purple edge** signals an abnormal component.

■ *VCV mode only. Switch to VCV first if another mode is active.*

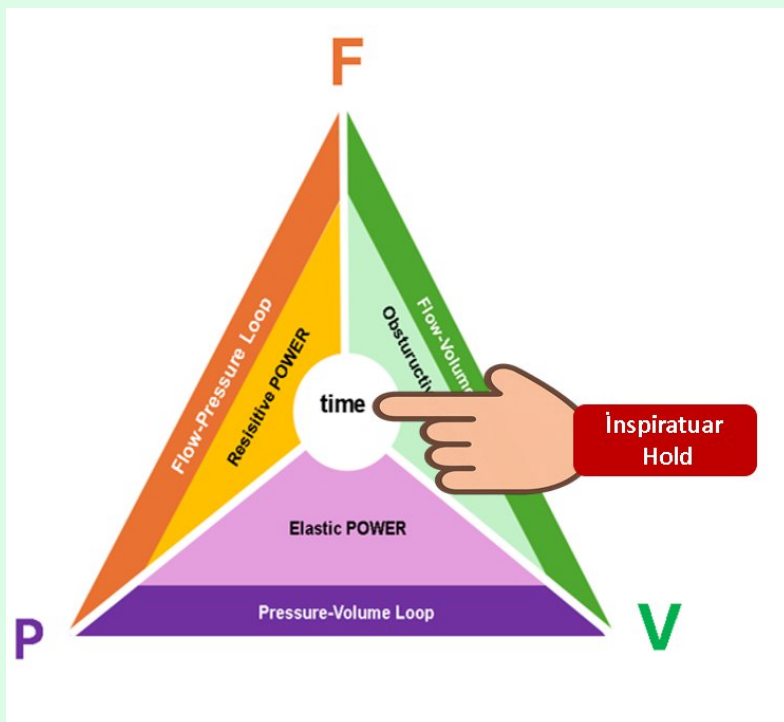

Click TIME → Insp. Hold

3

Click the pyramid edges — observe the loop graphics

→ Click the **left edge** → **F–P loop**: resistive gradient (P_{peak}–P_{plat}).

→ Click the **right edge** → **F–V loop**: expiratory flow limitation and air-trapping pattern.

→ Click the **bottom edge** → **P–V loop**: compliance curve and elastic work area.

→ Click the same edge again to **close** the loop view.

■ *Any color other than purple on an edge indicates pathology in that mechanical component.*

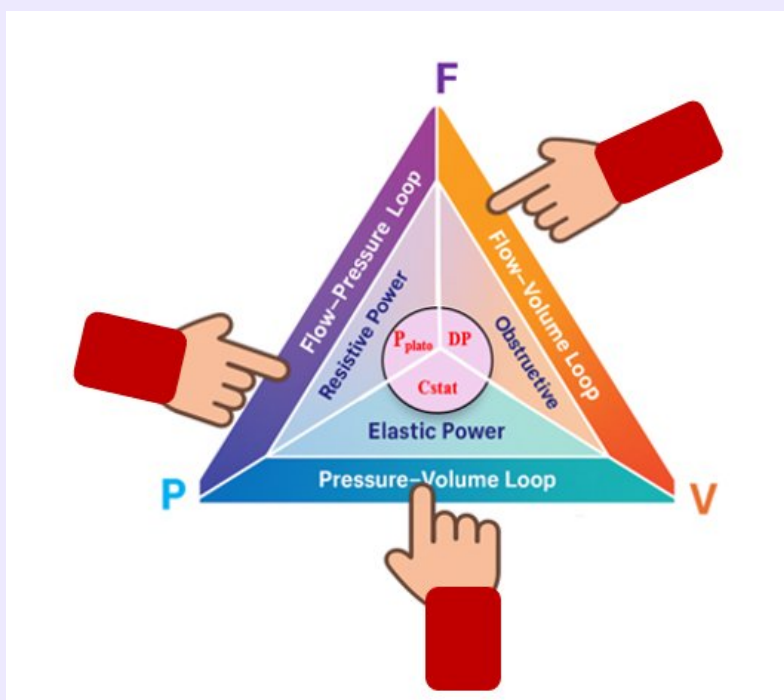

Click edges → loop views

4

Read and interpret the hold-derived metrics

→ After the hold, the parameter display and pyramid annotations show:

→ P_{plat} and DP appear at the **pyramid center**. MP_{res} on the left face, MP_{el} on the bottom face, C_{stat} below the base.

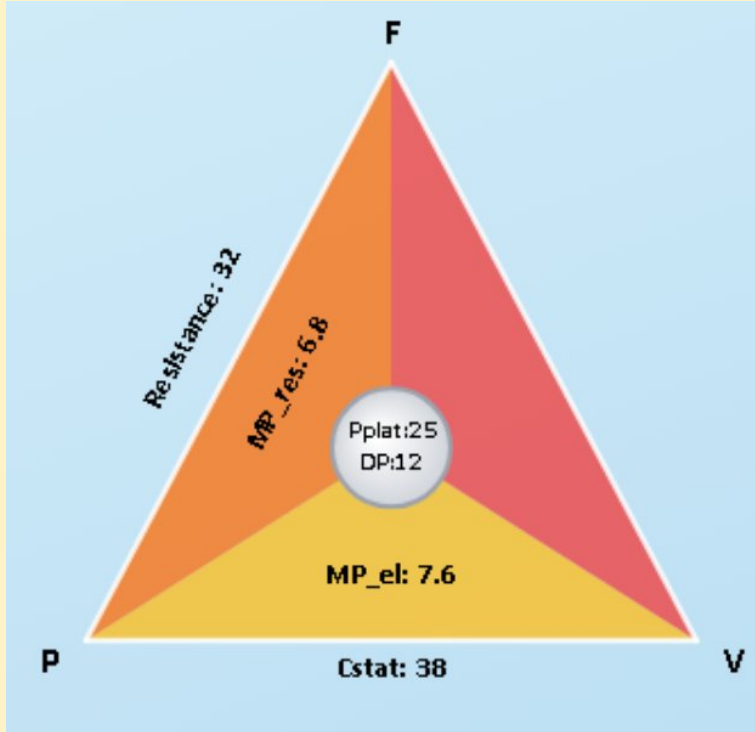

Hold-derived metrics on pyramid

Metric	What it tells you	Normal / Target	Action if abnormal
**P_{plat}**	Plateau pressure — elastic recoil	≤30 cmH_2O	Elevated: reduce VT, check compliance
**DP**	P_{plat}–PEEP — lung strain	≤15 cmH_2O	Elevated: reduce VT or titrate PEEP
**C_{stat}**	Static compliance — lung stiffness	35–50 mL/cmH_2O	Low: bottom edge red — PEEP trial
**MP_{el}**	Elastic mechanical power	<12 J/min	Elevated: VILI risk — lower VT or RR
**MP_{res}**	Resistive mechanical power	<5 J/min	Elevated: bronchodilator? Secretion clearance?
**PAI**	MP_{res}/MP_{el} — dominant phenotype	0.20–0.50	>1.0: resistive · <0.20: elastic
**τ_{est}**	Expiratory time constant	0.5–0.8 s	>1.2 s: auto-PEEP risk — RR↓ or I:E↑

5

Expiratory Hold — measure auto-PEEP

→ Click the **right inner zone** of the pyramid — the inner area of the F–V edge (obstructive zone, right side).

→ An expiratory hold triggers; **auto-PEEP** and $PEEP_{tot}$ are displayed.

→ The right edge deepens in color when auto-PEEP is present.

→ Click the same area again to **release** the hold.

■ *When auto-PEEP > 0, tau_est is automatically recalculated — watch the updated value on the display.*

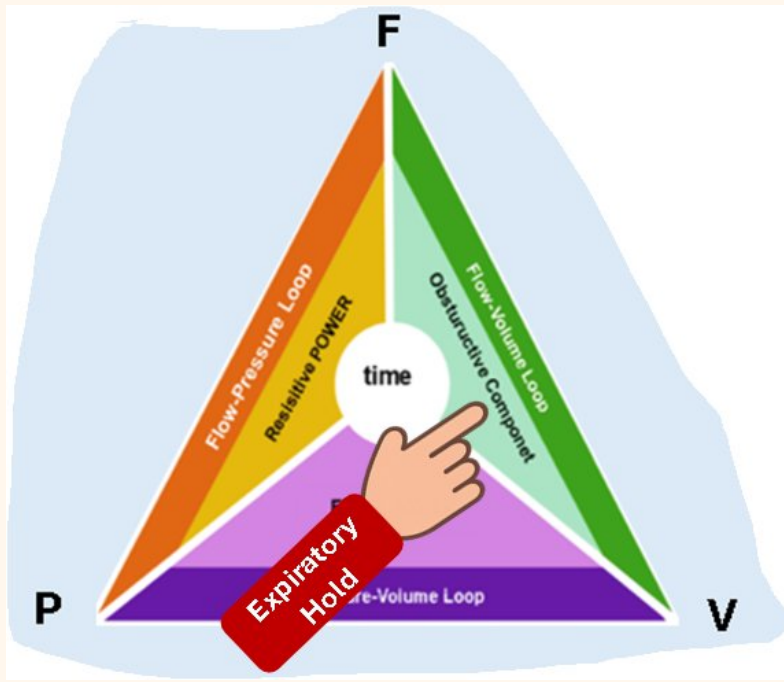

Click right inner zone → Exp. Hold

Quick Decision Summary

Pyramid Pattern	Likely Diagnosis	Action
**Bottom edge red + C_{stat} low**	ARDS / fibrosis / pneumothorax	Reduce VT · titrate PEEP · monitor DP
**Left edge red + P_{peak}–P_{plat} high**	Bronchospasm / secretions	Bronchodilator · suction · reduce RR
**Right edge red + τ_{est} >1.2 s**	COPD / air trapping / auto-PEEP	Increase I:E · reduce RR · extrinsic PEEP
**All edges purple · PAI 0.2–0.5**	Balanced mechanics	Maintain current settings
**PAI > 1.0**	Resistive dominant	Address airway obstruction first
**PAI < 0.20**	Elastic dominant	Optimize PEEP and VT for compliance

■ **Disclaimer: Educational and research tool only. All values are model-based and do not reflect patient-derived measurements. Must not be used for direct patient care. τ_{est} and PAI require prospective clinical validation.** ***Efe S, 2025 — Bursa Uludag University ICU.***
