# Supplementary material for: Respiratory pyramid interface (RPI): a conceptual three-dimensional pyramid model for visualization of lung mechanics during mechanical ventilation
Source: Front Physiol. 2026 Jul 29;17:1846417. doi: 10.3389/fphys.2026.1846417 (PMC13461443; doi:10.3389/fphys.2026.1846417)
Supplement: Supplementary file 2 [file Table1.docx]

**Table 1**. Simulated clinical scenarios integrated into the Respiratory Pyramid Interface.

| **Scenario** | **Ppeak** | **Pplat** | **PEEP** | **Cstat** | **Resist.** | **auto-PEEP** | **MP_el** | **MP_res** | **PAI** | **τ_est (s)** | **Pyramid Pattern** | |
| --- | --- | --- | --- | --- | --- | --- | --- | --- | --- | --- | --- | --- |
| **Normal** | 22 | 18 | 5 | 35 | 4 | 0 | 4.2 | 1.8 | 0.43 | 0.34 | Balanced elastic/resistive zones; all regions purple |  |
| **ARDS** | 39 | 32 | 5 | 21 | 7 | 0 | 17.5 | 2.4 | 0.14 | 0.26 | Elastic zone red; resistive mild; obstructive absent |  |
| **Mixed**  **(ARDS + ↑R)** | 44 | 29 | 8 | 20 | 15 | 0 | 12,8 | 5,3 | 0.41 | 0.57 | Both elastic and resistive zones elevated |  |
| **Bronchospasm** | 38 | 22 | 5 | 26 | 24 | 3 | 5.1 | 9.2 | 1.80 | 1.04 | Resistive zone red; F-V loop shows expiratory limitation |  |
| **COPD / auto-PEEP** | 47 | 25 | 5 | 38 | 32 | 9 | 7.6 | 6.8 | 0.89 | 1.10 | Resistive + obstructive zones red; τ_est markedly prolonged |  |
| **Obesity / Restrict.** | 30 | 26 | 8 | 23 | 9 | 0 | 9.8 | 3.2 | 0.33 | 0.24 | Elastic zone orange; resistive mild |  |
| **Pulm. Fibrosis** | 42 | 36 | 6 | 13 | 8 | 0 | 19.8 | 2.1 | 0.11 | 0.20 | Elastic zone dark red; critical DP; VILI alert |  |
| **Pneumothorax** | 48 | 40 | 5 | 8 | 9 | 0 | 24.5 | 2.8 | 0.11 | 0.23 | Acute elastic collapse; P-V loop narrows dramatically |  |
| **Card. Pul. Edema** | 36 | 30 | 12 | 23 | 7 | 0 | 12.4 | 2.2 | 0.18 | 0.33 | Elastic zone orange; PEEP titration shifts pattern toward normal |  |
| **Fat Embolism / PE** | 38 | 28 | 8 | 22 | 11 | 2 | 15.6 | 3.8 | 0.24 | 0.50 | Mixed elevation; mild obstructive; wide F-P loop |  |
| **Pregnancy (3rd T)** | 26 | 21 | 5 | 30 | 6 | 0 | 8.2 | 1.9 | 0.23 | 0.34 | Mildly reduced compliance; low-normal pattern |  |

**Ppeak:** peak airway pressure (cmH₂O); **Pplat:** plateau pressure (cmH₂O); **PEEP:** positive end-expiratory pressure (cmH₂O); **Cstat:** static compliance (mL/cmH₂O); **Resist.:** resistive pressure gradient (Ppeak − Pplat, cmH₂O); **auto-PEEP:** dynamic hyperinflation (cmH₂O); **MP_el:** elastic mechanical power (J/min); **MP_res:** resistive mechanical power (J/min); **PAI:** Pyramid Asymmetry Index (MP_res/MP_el); **τ_est:** pressure-derived expiratory time constant (seconds).
