# Supplementary material for: Respiratory pyramid interface (RPI): a conceptual three-dimensional pyramid model for visualization of lung mechanics during mechanical ventilation
Source: Front Physiol. 2026 Jul 29;17:1846417. doi: 10.3389/fphys.2026.1846417 (PMC13461443; doi:10.3389/fphys.2026.1846417)
Supplement: Supplementary file 3 [file Table2.docx]

**Supplementary File S2**

**Algorithmic Generation of the Pyramid Visualization Pseudocode and Flowchart**

This file specifies, in implementation-independent form, how the Respiratory Pyramid Interface (RPI) renders its visualization from routinely available ventilator parameters. It corresponds directly to the client-side HTML5/JavaScript source provided in Supplementary File S1. The pyramid uses a **fixed isosceles geometry**; quantitative information is encoded through **regional colour gradients** (for the three mechanical-power components) and through **numerical labels** (for all reported magnitudes and for the dimensionless indices PAI and τ_est). Edge lengths and vertex positions do not vary between patients.

**1. Pseudocode**

The following pseudocode reproduces the rendering pipeline of Supplementary File S1.

```
INPUT:  P_peak, P_plat, PEEP_set, PEEP_tot, V_T (mL), RR, T_insp
OUTPUT: pyramid rendered on a fixed 320 x 365 px canvas

# ---- Fixed geometry (identical for every patient) ----
F <- (160,  22)          # apex       : Flow
P <- ( 26, 276)          # base-left  : Pressure
V <- (294, 276)          # base-right : Volume
C <- (F + P + V) / 3     # centroid: shared interior node + hold control
# lateral edges P-F and F-V = 287 px; base edge P-V = 268 px (constant)

# ---- Step 1: continuous mechanical-power components (Eq. 2-3) ----
k         <- 0.098 * RR * (V_T / 1000)        # cmH2O.L -> J.min^-1 scaling
MP_el     <- k * (P_plat   - PEEP_tot)        # elastic   (Eq. 3)
MP_res    <- k * (P_peak   - P_plat)          # resistive (Eq. 2)
auto_PEEP <- PEEP_tot - PEEP_set
MP_aPEEP  <- k * auto_PEEP                     # obstructive (auto-PEEP-derived)

# ---- Step 2: dimensionless descriptors (NOT mapped to geometry/colour) ----
PAI     <- MP_res / MP_el                      # Eq. 4 ; continuous, no cut-offs
tau_est <- T_insp * PAI                        # Eq. 6

# ---- Step 3: discretize each MP component to a severity level 0..3 ----
sev_el(x):    if x < 13  -> 0 ; elif x <= 15 -> 1 ; elif x <= 17 -> 2 ; else -> 3
sev_res(x):   if x < 3   -> 0 ; elif x <= 5  -> 1 ; elif x <= 7  -> 2 ; else -> 3
sev_aPEEP(a): if a == 0  -> 0 ; elif a <= 4  -> 1 ; elif a <= 8  -> 2 ; else -> 3

PALETTE <- ["#c084fc", "#fbbf24", "#f97316", "#ef4444"]   # lavender->yellow->orange->red
# level 0 = lavender = clinically normal state for every component

# severity may be supplied directly by a teaching scenario; otherwise computed:
s_el     <- scenario.elS  if defined  else  sev_el(MP_el)
s_res    <- scenario.rsS  if defined  else  sev_res(MP_res)
s_obstr  <- scenario.obS  if defined  else  sev_aPEEP(auto_PEEP)

# ---- Step 4: fill the three FIXED sub-triangles ----
fill_triangle(P, V, C, colour = PALETTE[s_el],    alpha = 0.80)   # base  -> MP_el
fill_triangle(F, P, C, colour = PALETTE[s_res],   alpha = 0.80)   # left  -> MP_res
fill_triangle(F, V, C, colour = PALETTE[s_obstr], alpha = 0.80)   # right -> auto-PEEP

# ---- Step 5: stroke the three edges (user-selected edge highlighted cyan) ----
stroke_edge(F, P) ; stroke_edge(P, V) ; stroke_edge(F, V)

# ---- Step 6: numerical / descriptor overlays (text, not geometric) ----
label_at(centroid(P,V,C), "MP_el = "  + MP_el)
label_at(centroid(F,P,C), "MP_res = " + MP_res)
if auto_PEEP >= 1:  label_at(centroid(F,V,C), "MP_autoPEEP = " + MP_aPEEP)
during hold:        label_at(C, "Pplat / DP / Cstat")
display_field("PAI", PAI)            # adjacent numerical field, continuous
display_field("tau_est", tau_est)    # adjacent numerical field, continuous
```

**2. Severity-threshold reference**

Each mechanical-power component is mapped to one of four severity levels by the fixed thresholds below. PAI and τ_est are not discretized and not colour-mapped; they are reported as continuous numerical values.

| **Sub-triangle (edge)** | **Component** | **s = 0 (lavender)** | **s = 1 (yellow)** | **s = 2 (orange)** | **s = 3 (red)** |
| --- | --- | --- | --- | --- | --- |
| Base (P–V) | MP_el (J·min⁻¹) | < 13 | 13–15 | > 15–17 | > 17 |
| Left (P–F) | MP_res (J·min⁻¹) | < 3 | 3–5 | > 5–7 | > 7 |
| Right (F–V) | auto-PEEP (cmH₂O) | 0 | 1–4 | 5–8 | > 8 |

**3. Flowchart**

The rendering pipeline proceeds through the following ordered steps.

```
   +---------------------------------------------+
   | INPUT                                       |
   | Ppeak, Pplat, PEEP_set, PEEP_tot,           |
   | VT, RR, T_insp                              |
   +----------------------+----------------------+
                          v
   +---------------------------------------------+
   | STEP 1 -- continuous values                 |
   | k, MP_el, MP_res, auto-PEEP, MP_auto-PEEP   |
   +----------------------+----------------------+
                          v
   +---------------------------------------------+
   | STEP 2 -- dimensionless descriptors         |
   | PAI = MP_res/MP_el ; tau_est = T_insp x PAI |
   | (reported numerically; NOT geometric)       |
   +----------------------+----------------------+
                          v
   +---------------------------------------------+
   | STEP 3 -- discretize the THREE MP           |
   | components to severity s in {0,1,2,3}       |
   | via fixed thresholds                        |
   | (PAI / tau_est are NOT binned)              |
   +----------------------+----------------------+
                          v
   +---------------------------------------------+
   | STEP 4 -- index the 4-level palette         |
   | lavender / yellow / orange / red            |
   +----------------------+----------------------+
                          v
   +---------------------------------------------+
   | STEP 5 -- fill the 3 FIXED sub-triangles    |
   | base = MP_el, left = MP_res,                |
   | right = auto-PEEP   (geometry constant)     |
   +----------------------+----------------------+
                          v
   +---------------------------------------------+
   | STEP 6 -- stroke edges + overlay numerical  |
   | labels; show PAI and tau_est as continuous  |
   | descriptors in adjacent fields              |
   +---------------------------------------------+
```

**4. Reference rendering constants**

The constants below, together with the six steps above, fully specify the visualization independently of the source code in Supplementary File S1.

| **Element** | **Value** |
| --- | --- |
| Canvas size | 320 × 365 logical px |
| Apex F (Flow) | (160, 22) |
| Base-left P (Pressure) | (26, 276) |
| Base-right V (Volume) | (294, 276) |
| Centroid C | (160, 191) |
| Lateral edges (P–F, F–V) | 287 px each |
| Base edge (P–V) | 268 px |
| Region fill opacity | 0.80 |
| Palette | #c084fc (lavender), #fbbf24 (yellow), #f97316 (orange), #ef4444 (red) |
| Unit-conversion constant | 0.098 (cmH₂O·L → J) |
