# Supplementary material for: Respiratory pyramid interface (RPI): a conceptual three-dimensional pyramid model for visualization of lung mechanics during mechanical ventilation
Source: Front Physiol. 2026 Jul 29;17:1846417. doi: 10.3389/fphys.2026.1846417 (PMC13461443; doi:10.3389/fphys.2026.1846417)
Supplement: Supplementary file 4 [file DataSheet1.zip › RPI_Simulator_S1.html]

Respiratory Pyramid Interface (RPI): A Three-Dimensional Pyramid Model for Visualization of Lung Mechanics During Mechanical Ventilation


Respiratory Pyramid Interface (RPI): A Three-Dimensional Pyramid Model for Visualization of Lung Mechanics During Mechanical Ventilation

Serdar Efe, MD · Department of Internal Medicine, Intensive Care Unit · Bursa Uludağ University, Bursa, Turkey

Patent pending: PCT/TR2025/052098 — Solunum Paneli Arayüzü Karar Destek Sistemi

SUPPLEMENTARY FILE S1

Interactive Simulation

Peer Review Version

⚕️ Educational & Research Tool Only
Values are model-based and do not replace clinical assessment. Not validated for direct patient care.

11 clinical scenarios
VCV · PCV · APRV · Bilevel · Bilevel-VG
τ\_est · PAI · Hold maneuvers

🫁 Normal

⚠️ ARDS

🔄 Prone
💉 ECMO

🔀 Mixed
🔴 Bronchospasm
🌀 COPD
📊 Obesity
🫧 Fibrosis
🚨 Pneumothorax
💧 Card.Edema
🩸 Fat Emb.
🤰 Pregnancy
↺ Reset
🏥 Patient Entry
🎓 Quiz

1:1
1:1.5
1:2
1:2.5
1:3
1:4

🏥 PATIENT PARAMETERS
Parametreye dokun → klavyeden gir

Ppeak

22

cmH₂O

Pplat

18

cmH₂O

PEEP

5

cmH₂O

VT

450

mL

RR

16

/min

auto-PEEP

0

cmH₂O

FiO₂

60

%

I:E

1:1

1:1.5

1:2

1:2.5

1:3

1:4

−

—

+

7
8
9
4
5
6
1
2
3
0
⌫

✓ Uygula

🎓 CLINICAL QUIZ
0 / 0

Loading question...

Next Question →

VCV
PCV
APRV
Bilevel
Bilevel-VG

ALARM

P

F

V

Loop

P peak

22

VT_insp_

450

VT_eksp_

440

I:E

1:1.5

Resp. Rate

16

PAI (res/el)

—

τ\_est (s)

—


FiO₂

%60

Volume

450

T\_insp

1.1

PEEP

5

Insp. Pause

Off

Trigger

2

Rate

16

⟳

FiO₂

%60

Psupp

16

T\_insp

1.2

PEEP

5

Trigger

2

Rate

16

⟳

FiO₂

%60

P\_high

28

P\_low

5

T\_high

4.0

T\_low

0.5

⟳

FiO₂

%60

P\_high

22

P\_low

8

T\_high

1.5

T\_low

3.0

⟳

FiO₂

%60

P\_high

22

P\_low

8

T\_high

1.5

T\_low

3.0

VT target

450

⟳

⚡ HL7 CANLI

⚙ Ayarlar

⏸ Not connected — simulation mode

CANLI

IP ADDRESS

PORT

BAĞLANTI TÜRÜ

WebSocket (ws://)
HTTP Polling

▶ Başlat

⏹ Durdur

MANUEL HL7 TEST — Bir HL7 mesajı yapıştırıp test edebilirsiniz

🧪 Test Et

Desteklenen kodlar: VENT-PPEAK, VENT-PPLAT, VENT-PEEP, VENT-VTI, VENT-VTE, VENT-RR, VENT-TINSP, PIP, RR, VTE, VTI, PEEP, TI ve LOINC kodları (20077-4, 76531-4, 20060-0...)

📖 Quick-Start Guide — Supplementary File S1
▼

① Select Scenario / Patient Entry

Click a scenario button (Normal, ARDS, COPD, Bronchospasm…) or use **🏥 Patient Entry** to enter real bedside values. Confirm **VCV mode** for hold maneuvers.

② Click TIME → Inspiratory Hold (5 sec)

Click the **TIME** label at the inner center of the pyramid. Waveform freezes at end-inspiration. P_plat_, DP, C_stat_, MP_el_, MP_res_, PAI, τ_est_ are displayed. **Any non-purple edge = pathology.**

③ Click Edges → Loop Graphics

**Left edge** → F–P loop (Resistive) · **Right edge** → F–V loop (Obstructive) · **Bottom edge** → P–V loop (Elastic). Click again to close.

④ Right Inner Zone → Expiratory Hold

Click the **right inner zone** (inside F–V edge) to trigger expiratory hold. auto-PEEP and PEEP_tot_ are displayed. τ_est_ recalculates automatically.

| Metric | Normal | Action if abnormal | Pyramid Pattern → Diagnosis |
| --- | --- | --- | --- |
| τ_est_ | 0.5–0.8 s | >1.2 s: ↓RR or ↑I:E | Right edge red → COPD / air trapping |
| PAI | 0.20–0.50 | >1.0: resistive · <0.20: elastic | Left edge red → Bronchospasm |
| DP | ≤15 cmH₂O | ↓VT or ↑PEEP | Bottom edge red → ARDS / Fibrosis |
| C_stat_ | 35–50 mL/cmH₂O | PEEP trial | All edges purple → Balanced |

**⚠️ Disclaimer:** Educational and research tool only. Values are model-based. Must not be used for direct patient care.
τ_est_ and PAI require prospective clinical validation. Patent pending: PCT/TR2025/052098.
*Efe S, 2025 — Bursa Uludağ University ICU.*
